# Establishment and optimization of a system for the detection of *Candida albicans* based on enzymatic recombinase amplification and CRISPR/Cas12a system

**DOI:** 10.1128/spectrum.00268-25

**Published:** 2025-03-31

**Authors:** Xiaotong Zeng, Qiuyang Jiang, Fo Yang, Qianlin Wu, Tingyao lyu, Qi Zhang, Jin Wang, Feng Li, Dayong Xu

**Affiliations:** 1 Anhui Province Key Laboratory of Pollutant Sensitive Materials and Environmental Remediation, Huaibei Normal University 58286, Huaibei, Anhui, China; 2 School of Life Sciences, Huaibei Normal University 58286, Huaibei, Anhui, China; 3 Huaibei People’s Hospital, Huaibei, Anhui, China; 4 Department of Clinical Laboratory, Shenzhen Institute of Translational Medicine, The First Affiliated Hospital of Shenzhen University, Shenzhen Second People’s Hospital 47890, Shenzhen, Guangdong, China; 5 Tolo Biotech Co., Ltd, Wuxi, Jiangsu, China; Children's National Hospital, George Washington University, Washington, DC, USA

**Keywords:** *Candida albicans*, ERA, CRISPR/Cas12a

## Abstract

Invasive candidiasis is a fungal infection caused by various pathogenic yeasts, with *Candida albicans* as the predominant pathogen. Traditional culturing and identification methods for *C. albicans* are slow, requiring several days to weeks to produce results, which hampers rapid diagnosis. In this study, we proposed three amplification methods to combine with CRISPR/Cas12a and selected the enzymatic recombinase amplification (ERA) and CRISPR/Cas12a two-step method for the detection of *C. albicans* in terms of sensitivity, and then the two-step method was optimized to a temperature-controlled one-step method for the detection of *C. albicans* by enzymatic recombinase amplification (ERA)-CRISPR/Cas12a. The temperature-controlled system employs a combination of liquid and solid paraffin wax to maintain the desired melting point, thus facilitating spatial separation of the ERA amplification system from the CRISPR/Cas12a detection system within a single tube. After a reaction at 37°C, the temperature is raised to 45°C, melting the wax and allowing the amplification system to merge with the detection system, initiating the reaction. This one**-**step detection platform simplifies and expedites the procedure, achieving a sensitivity level on par with that of two**-**step methods. The reaction completes in about 30 minutes, detecting as little as 100 ag/µL of genomic DNA from *C. albicans* pure cultures. It shows high specificity and resistance to clinical nucleic acid interference, without cross**-**reactivity. Additionally, the method eliminates the need to open the reaction tube, effectively preventing aerosol contamination and providing a stable, thus offering a new tool for the rapid clinical diagnosis of *C. albicans*.

**IMPORTANCE:**

This study established a two-step method through optimization, compared its sensitivity, and then combined the specific detection capabilities of ERA and CRISPR/Cas12a. Furthermore, a one-step method was developed based on the two-step method, creating a one-step system for the detection of *Candida albicans*. This system does not require the lid to be opened during the reaction process, reducing aerosol contamination and minimizing the risk of false positives. This method does not require advanced instruments or equipment and shows strong specificity without being affected by other pathogens. It can serve as a new method for the detection of *Candida albicans* and has significant practical application prospects.

## INTRODUCTION

Invasive candidiasis (IC) is a clinically prevalent fungal infection that is often observed in immunocompromised patients and is characterized by a high incidence, elevated mortality rates, and substantial treatment costs. The pathogens most commonly implicated in IC are *C. albicans*, *C. glabrata*, *C. tropicalis*, *C. parapsilosis*, and *C. krusei*, with *C. albicans* responsible for approximately 65%–70% of cases (1). *C. albicans* is an opportunistic fungal pathogen that is prevalent both in the environment and in the body (2). Normally, *C. albicans* exists symbiotically with the human host and is not pathogenic. However, if the immune system is weakened or the microbial balance in areas such as the vaginal or oral mucosa is disrupted, *C. albicans* can proliferate, causing various infections (3, 4). Blood culture, currently the gold standard for diagnosing *C. albicans* infections, involves collecting samples from potential infection sites such as blood, urine, and respiratory secretions and culturing them on specific media. During incubation, *C. albicans* forms characteristic colonies that facilitate preliminary identification based on morphological features (5). Although this method is highly accurate, it is plagued by a prolonged incubation period and limited sensitivity, which hampers its diagnostic utility. Other diagnostic methods, such as mannan/anti**-**mannan immunoglobulin G detection, (1,3)**-**β**-**d**-**glucan testing (G**-**test) (6), and quantitative PCR (qPCR) assays (7), are used in some laboratories but have not been extensively evaluated in cases of deep-seated candidiasis with negative blood cultures (8). These techniques each have drawbacks, including extended diagnostic times and suboptimal specificity or sensitivity. Consequently, the early diagnosis and development of novel diagnostic technologies that improve sensitivity and specificity are crucial.

In recent years, the CRISPR/Cas system (clustered regularly interspaced short palindromic repeats and associated proteins) has demonstrated significant advantages for point-of-care testing (POCT) due to its short detection time and high specificity (9). Under the guidance of CRISPR RNA (crRNA), this system specifically recognizes target DNA, activating the Cas12a protein from Lachnospiraceae bacterium (LbCas12a), which exhibits cis- and trans-cleavage activities (10, 11). These activities enable the specific cleavage of double-stranded DNA (dsDNA) targets and the nonspecific cleavage of single**-**stranded DNA (ssDNA) probes, translating nucleic acid target signals into detectable outputs observable either by instrumentation or visually (12). This mechanism facilitates integration with nucleic acid amplification systems such as polymerase chain reaction (PCR)—a heat-cycling process requiring 1–2 hours and specialized equipment—and isothermal amplification methods like loop-mediated isothermal amplification (LAMP), recombinase polymerase amplification (RPA), and enzyme**-**assisted isothermal amplification (ERA). Isothermal amplification. Therefore, the use of a nucleic acid amplification system with high sensitivity, coupled with a CRISPR/Cas detection system with high specificity, better meets the demands of POCT (13, 14). Examples include CRISPR/Cas detection based on PCR (15), CRISPR/Cas detection based on loop-mediated isothermal amplification (16, 17), CRISPR/Cas detection based on RPA (18, 19), and CRISPR/Cas detection based on enzyme**-**driven isothermal amplification (20, 21).

However, most of these methods require two separate reaction steps, which may result in aerosol contamination during reagent transfer or lid opening, increasing the likelihood of false positives. Alternatively, directly mixing the amplification and detection systems for simultaneous amplification and detection may lead to a rapid decrease in sensitivity or an extended reaction time (22). This is due to the fact that the template and small amplification fragments in the amplification system can activate both the cis**-** and trans**-**cutting activities of the CRISPR/Cas12a enzyme in the detection system. The cis**-**cutting activity leads to the cleavage of the template and amplification fragments, while the trans-cutting activity results in the cleavage of the corresponding single-stranded primers of the amplification fragments, thereby significantly weakening the amplification efficiency (23, 24). To overcome these challenges, several single-tube reaction systems employing spatial separation within the tube or modified crRNA processing have been developed. For example, the unique structure of PCR tubes has been used to separate amplification reagents and CRISPR/Cas12a reagents by placing them in the tube cap and tube base, respectively, allowing addition in a single step (22). During the initial phase of amplification, the viscosity of glycerol separates the amplification system from the CRISPR/Cas12a system within the tube (25). Innovative methods include a photo**-**activated CRISPR**-**Cas12 system utilizing a 6-nitropiperonylmethoxy (NPOM)-modified crRNA. The NPOM**-**caged crRNA inhibits Watson-Crick base pairing between crRNA and target DNA, silencing CRISPR-Cas12 activity until UV-induced uncaging restores it (26). Another approach uses a photo-cleavable linker (PC-linker) modified silencing oligonucleotide that hybridizes with crRNA, blocking the recognition and cleavage functions of the CRISPR system. Upon completion of nucleic acid amplification, UV irradiation cleaves the silencing oligonucleotide, restoring CRISPR cleavage activity. This method effectively separates nucleic acid amplification from CRISPR reactions in a closed, single-tube, high-sensitivity setting (27). These approaches aim to mitigate interference between nucleic acid amplification and the CRISPR system but may require complex system optimization and could encounter issues with reproducibility and stability. Consequently, a temperature-controlled, automated one-step ERA-CRISPR/Cas12a method has been developed, offering rapid, simple, and relatively low-cost detection of *C. albicans*.

In this study, initial optimization of specific conditions was conducted, including the selection of primers for the amplification system and the optimization of LbCas12a enzyme and crRNA concentrations in the detection system. Subsequently, three amplification methods—PCR, ERA, and LAMP (one variable**-**temperature and two isothermal methods)—were integrated with the CRISPR/Cas system in a two-step format, with the sensitivity (LOD) of each method compared. Based on these results, ERA was selected as the amplification method for *C. albicans* nucleic acid. Building on the two-step process, a temperature**-**controlled, one**-**step ERA**-**CRISPR/Cas12a assay was developed. In this setup, the ERA amplification system and the CRISPR detection system were combined within a single tube, separated by wax (ERA in the upper layer, CRISPR in the lower layer). We reviewed the data and found that ERA was amplified at 37°C (28), and LbCas12a was active at 24°C–48°C (29). Using solid wax alone for separation posed a risk of a melting point too high for the optimal operating temperature of LbCas12a. Therefore, a wax mixture of liquid and solid paraffin was created to achieve a melting point of around 45°C, allowing both for wax melting and attainment of the LbCas12a operational temperature. This configuration enabled ERA amplification to proceed independently at 37°C, followed by heating to 45°C, at which point the wax melted, allowing the ERA reagents to merge with the CRISPR detection system for *C. albicans* detection. This process did not require centrifugation or tube opening, and detection was performed using a fluorescent qPCR instrument programmed for the reaction. The specificity and clinical sample detection capability of this platform were further evaluated, establishing it as a highly sensitive, specific, and convenient single-tube platform for *C. albicans* detection.

## MATERIALS AND METHODS

### Materials and reagents

Recombinant plasmids, primers, and Target Antisense Oligo were synthesized by Sangon Biotechnology Co., Ltd. (Shanghai, China), with sequence information provided in Table S1. The 2 × MegaPfu Premix, 2 × Tolo Fluorescent LAMP Premix, Cas12a High Yield CrRNA Synthesis and Purification Kit, LbCas12a (Cpf1) Nuclease, and HOLMES ssDNA reporter (FAM) were purchased from Tolo Biotech Co., Ltd. (Shanghai, China). The basic nucleic acid amplification kit (ERA method) was obtained from GenDx Biotech Co., Ltd. (Suzhou, China). Liquid paraffin, solid paraffin, and other chemical reagents were sourced from Sinopharm Chemical Reagent Co., Ltd. (Shanghai, China). RNase-free materials were supplied by Sangon Biotechnology Co., Ltd. (Shanghai, China). *C. albicans* (ATCC5314), *Escherichia coli*, *Staphylococcus aureus*, *Salmonella*, and *Aspergillus fumigatus* were acquired from laboratory resources at Huaibei Normal University. *C. parapsilosis*, *C. tropicalis*, *C. viswanathii*, *C. guilliermondii*, and clinical samples of *C. albicans* were provided by Huaibei People’s Hospital.

### Recombinant plasmid construction and genomic DNA extraction

A recombinant pUC19 plasmid, containing the *C. albicans* internal transcribed spacer 2 (ITS2) gene sequence (GenBank accession number AF455531.1), was synthesized by Sangon Biotechnology Co., Ltd. (Shanghai, China). The plasmid was extracted using a plasmid extraction kit, and the nucleic acid concentration was measured via UV spectrophotometry. The plasmid concentration was converted to copy number using the formula: concentration (copies/µL) = [6.02 × 10^23^ × concentration (ng/µL) ×10^−9^]/[DNA length (bp) × 660]. The plasmid was then serially diluted 10-fold from 1 × 10^6^ copies/µL to 1 × 10° copies/µL. *C. albicans* was cultured in YPD medium at 30°C for 18 hours, and crude genomic DNA was extracted by the glass bead method, followed by serial 10-fold dilution from 10 pg/µL to 10 ag/µL. Genomic DNA was also extracted from additional strains, including *C. parapsilosis*, *C. tropicalis*, *C. Viswanathan*, *C. guilliermondii*, *E. coli*, *S. aureus*, *Salmonella*, and *Aspergillus fumigatus*. All nucleic acids were stored below −20°C for future use.

### Transcription and preparation of crRNA

A specific crRNA was designed targeting the *C. albicans* ITS2 gene and the Cas12a protospacer adjacent motif (PAM) recognition site (TTTV), using software available at www.rgenome.net/cas-designer/. Considering the cost, *in vitro* transcription was chosen for crRNA preparation, requiring the design of a Target Antisense Oligo corresponding to the crRNA. The Target Antisense Oligo was denatured at 95°C for 2 minutes and then slowly annealed to room temperature with the Cas12a Sense Oligo (included with the *in vitro* transcription kit) to generate the DNA transcription template. The transcription reaction mixture was incubated at 37°C for 4 hours, after which residual DNA templates were digested with DNase I. The transcription products were purified using magnetic beads provided with the kit. RNA concentrations were quantified using a microvolume spectrophotometer, and the purified RNA was stored at **−**80°C. All sequences are listed in Table S1.

### Primer design and screening

For the amplification of the *C. albicans* ITS2 gene, primer sets were designed to correspond to the selected crRNA targets using two distinct software tools: Primer Premier 5.0 for PCR and ERA, and Primer Explorer V5 for LAMP methods. Each method employed three distinct sets of primers to determine the most effective one via BLAST analysis for specificity confirmation.

PCR primer screening: The PCR protocol commenced with the assembly of reaction mixtures, consisting of 12.5 µL of 2 × MegaPfu Premix, 2 µL of 10 µM each forward and reverse primers, 2 µL of DNA template, and 8.5 µL of ddH_2_O. The amplification protocol was structured as follows: an initial denaturation at 95°C for 5 minutes, 35 cycles of 94°C for 30 seconds, 56°C for 30 seconds, and 72°C for 30 seconds, concluding with a final elongation at 72°C for 10 minutes. Gel electrophoresis of 1% agarose was utilized to assess the products from the primer trios.

ERA primer screening: This involved preparing a reaction mix with 20 µL of solvent, 2.5 µL of 10 µM each forward and reverse primers, 4 µL of DNA template, and 19 µL of ddH_2_O. The mixture was introduced into an eppendorf tube containing lyophilized enzyme, sealed with 2 µL of activator, and centrifuged briefly. Incubation proceeded in a metal bath at 37°C for 20 minutes, followed by a 5 minute stabilization at 56°C. The results were then evaluated using 1% agarose gel electrophoresis.

LAMP primer screening: The LAMP reaction consisted of 12.5 µL of 2 × Tolo Super LAMP Premix, 2.5 µL of 10 × LAMP Primers Mix, 0.5 µL of 50 × LAMP fluorescent dye, 2 µL of DNA template, and 7.5 µL of ddH_2_O. The comprehensive mix was then processed in a LightCycler96 system at 65°C, where fluorescence signals were continuously monitored to determine the effectiveness of the primer sets.

### Cis-cleavage assay and trans-cleavage assay

The cis-cleavage assay aimed to determine the specific dsDNA target cleavage by LbCas12a within the assay system. The reaction mixture, comprising LbCas12a and crRNA at a concentration of 250 nM, was supplemented with 2 µL of 10 × HOLMES Buffer, 2 µL of dsDNA target, and topped up with nuclease-free water to a total volume of 20 µL. After thorough mixing and centrifugation, the mixture was incubated at 37°C for 40 minutes and subsequently deactivated at 85°C for 5 minutes. The cleavage results were visualized on a 1% agarose gel.

The trans-cleavage assay evaluated the ability of LbCas12a to cleave a non-specific ssDNA probe, thereby demonstrating its trans-cleavage capability. The assay was designed with nine different experimental conditions to test the versatility of CRISPR/Cas12a trans-cleavage activity. Each reaction, containing LbCas12a, crRNA, and ssDNA probe at 250 nM, along with 2 µL of 10 × HOLMES Buffer and 2 µL of dsDNA target, was adjusted to a 20 µL final volume with nuclease-free water. The comprehensive reaction was then subjected to real-time fluorescence monitoring in a LightCycler96 system at 37°C.

### Optimization of CRISPR/Cas12a reaction system

To enhance the efficacy and cost-effectiveness of the CRISPR/Cas12a system, optimization experiments were carried out on the concentrations of ssDNA and the LbCas12a/crRNA complex. The reaction system, comprising 250 nM each of LbCas12a, crRNA, and ssDNA, also included 2 µL of 10 × HOLMES Buffer, 2 µL of dsDNA target, and nuclease-free water, totaling a volume of 20 µL. For ssDNA, various concentrations (100, 200, 300, 400, and 500 nM) were tested while maintaining other components constant. These reactions were conducted at 37°C using the LightCycler96 system to monitor fluorescence output.

Additionally, the ratio of LbCas12a to crRNA was adjusted by fixing the crRNA at 250 nM and altering the LbCas12a concentration between 100 nM and 300 nM. This adjustment aimed to determine the optimal enzyme to guide RNA ratio under constant conditions of other reaction components. These were assessed for fluorescence response in the same fluorescence detection system at 37°C.

### Two-step CRISPR/Cas12a fluorescence detection

The two-step fluorescence assay for CRISPR/Cas12a involved a reaction mixture containing 200 nM LbCas12a, 250 nM crRNA, and 200 nM ssDNA reporter probe, complemented with 2 µL of 10 × HOLMES Buffer and 2 µL of amplification product from PCR, ERA, or LAMP reactions. The total reaction volume was adjusted to 20 µL with nuclease**-**free water. This mixture was homogenized, briefly centrifuged, and then subjected to real-time fluorescence monitoring at 37°C in the LightCycler96 system.

### Construction of one-step ERA-CRISPR/Cas12a detection system

In constructing the one-step ERA**-**CRISPR/Cas12a system, a blended wax composed of varying ratios of liquid to solid paraffin (from 10:1 to 10:8) was utilized to find an optimal melting point that effectively separates the ERA and CRISPR reactions. The ERA system contained 20 µL of solvent, 2.5 µL each of forward and reverse primers (10 µM), 2 µL of activator, and 19 µL of ddH_2_O, mixed thoroughly and added to an eppendorf tube with lyophilized enzyme. The CRISPR detection mix, consisting of 200 nM each of LbCas12a and ssDNA, 250 nM crRNA, and 2 µL of 10 × HOLMES Buffer, was prepared to a 20 µL volume and layered beneath a sealing layer of blended wax in the tube. The ERA mixture, with an added 2 µL of DNA template, was placed atop the wax. The entire assembly was then placed in a real**-**time PCR system, maintaining the ERA phase at 37°C for 10 minutes and subsequent melting of the wax and initiation of the CRISPR reaction at 45°C.

### Clinical evaluation of one-step ERA-CRISPR/ Cas12a system

Clinical evaluation of the one**-**step ERA**-**CRISPR/Cas12a detection approach involved 21 saliva nucleic acid samples sourced from Huaibei People’s Hospital, confirmed negative for *C. albicans*. To simulate testing conditions, 18 of these samples were artificially spiked with *C. albicans* genomic material, creating a series of positive samples with concentrations ranging from 10 pg/µL to 100 ag/µL. The method was applied to these samples, alongside three unmodified negative samples and three nuclease**-**free water controls, to assess the system’s efficacy under clinical conditions.

## RESULTS AND DISCUSSION

### Screening of primers for PCR, ERA, and LAMP

Primer screening was conducted for three amplification methods: PCR, ERA, and LAMP. Different approaches were utilized to analyze the results due to the varied principles of amplification. Both PCR and ERA required two complementary primers to amplify the target fragment, which was analyzed using agarose gel electrophoresis to identify the most suitable primer pairs. LAMP amplification, which requires four specific primers targeting six regions of the target gene, produced multiple fragments of varying sizes. Due to the complexity of the reaction products, which typically exhibit a ladder**-**like appearance on electrophoresis, a dye**-**based method was used for real**-**time fluorescence monitoring to select the optimal primer pairs.

**Fig 1:**
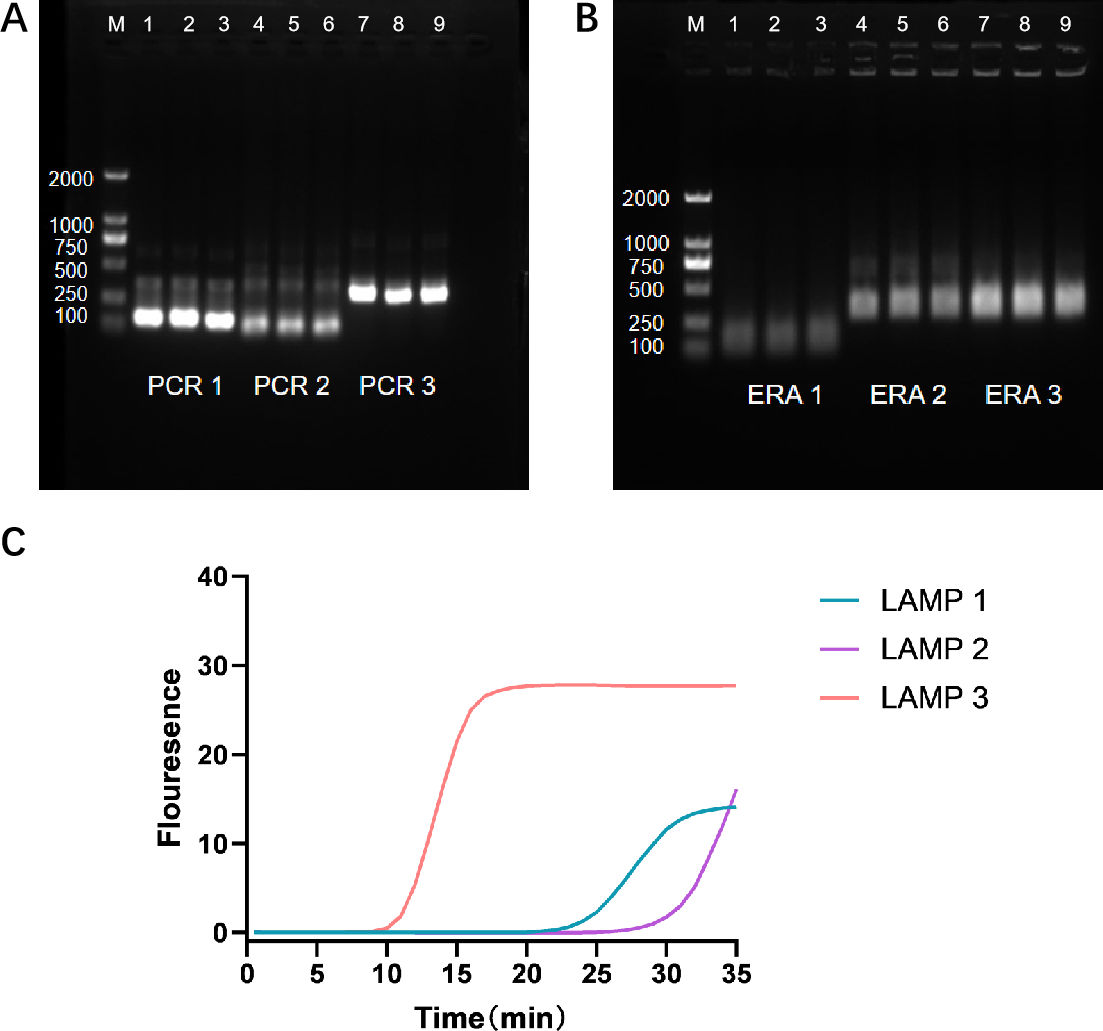
Selection of Optimal Primers for PCR, ERA, and LAMP. (**A**) Agarose gel image from PCR primer screening. (**B**) Agarose gel image from ERA primer screening. (**C**) Kinetic curves of fluorescence measurement from LAMP primer screening, displaying average results from three biological replicates.

As illustrated in Fig. 1A, PCR primer 3 showed a prominent band at the target position with minimal non**-**specific bands, indicating greater amplification efficiency compared to the other primer pairs. In Fig. 1B, ERA primer 3 also displayed a clear band at the target position, with relatively weak non**-**specific bands, suggesting it was more effective than the other pairs. The amplification curves from fluorescence detection in Fig. 1C indicated that LAMP primer 3 started to show fluorescence at 10 minutes and plateaued at 15 minutes, whereas the other primer pairs began to fluoresce after 20 minutes, demonstrating that LAMP primer 3 was more efficient. Consequently, PCR primer 3, ERA primer 3, and LAMP primer 3 were selected as the optimal primers for their respective methods.

### Validation of CRISPR/Cas12a cis-cleavage and trans-cleavage

**Fig 2:**
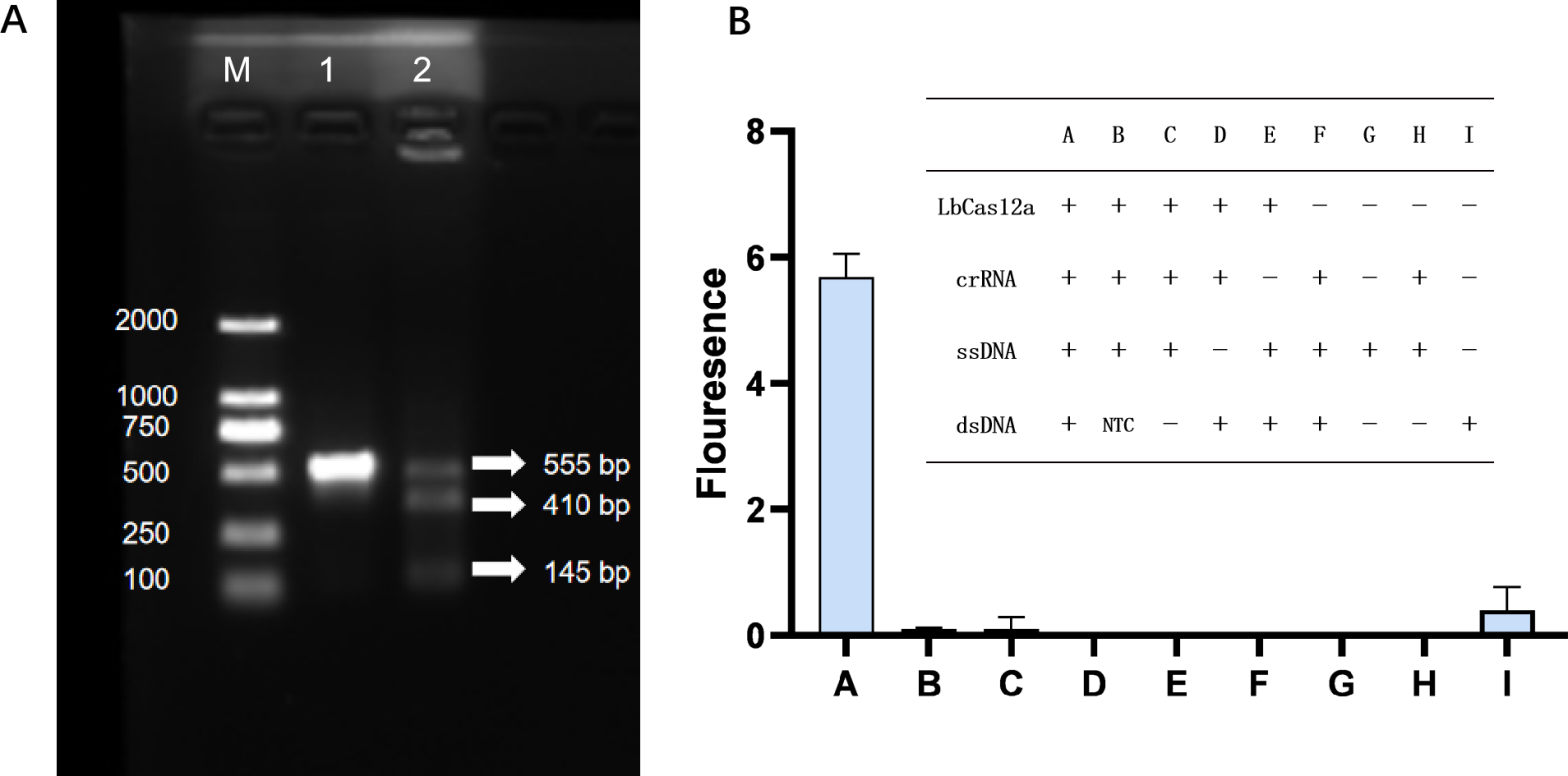
Validation of CRISPR/Cas12a cis-cleavage and trans-cleavage activities. (**A**) Validation of the cis-cleavage activity of LbCas12a. Lane M: DL2000 Marker; Lane 1: intact 555 bp target dsDNA; Lane 2: remaining target dsDNA and two cis-cleavage products, measuring 410 bp and 145 bp, respectively. (**B**) Validation of the trans-cleavage activity of LbCas12a. Group A: complete reaction components; Group B: negative control (NTC) using nuclease-free water instead of target DNA; Groups C–I: incomplete reaction components. Average results from three biological replicates are shown.

Primers ITS2**-**F and ITS2**-**R (Table S1) were used to amplify a 555 bp target fragment containing the PAM site via PCR. The PCR product was then introduced to the CRISPR/Cas12a detection system, which demonstrated that the target fragment was cleaved into two fragments of 410 bp and 145 bp (Fig. 2A), thus confirming the cis**-**cleavage activity of LbCas12a. Additionally, a report probe ssDNA

was incorporated into the system, and nine variable experimental sets (A**-**I) were established. Observations from Fig. 2B indicated that only Group A showed a strong fluorescence signal, while Groups B–I displayed negligible fluorescence. This finding validated the trans**-**cleavage activity of LbCas12a. It was also verified that forming a ternary complex with LbCas12a, crRNA, and the target fragment is essential to activate the enzyme and induce both cleavage activities.

### Optimization of the CRISPR/Cas12a-based fluorescence detection system

The kinetics of the CRISPR/Cas12a were examined to optimize the concentrations of ssDNA and the LbCas12a/crRNA complex, considering signal intensity, rapid plateau achievement, and cost**-**efficiency. A recombinant plasmid at a concentration of 1 × 10^6^ copies/µL served as the template for this analysis.

**Fig 3:**
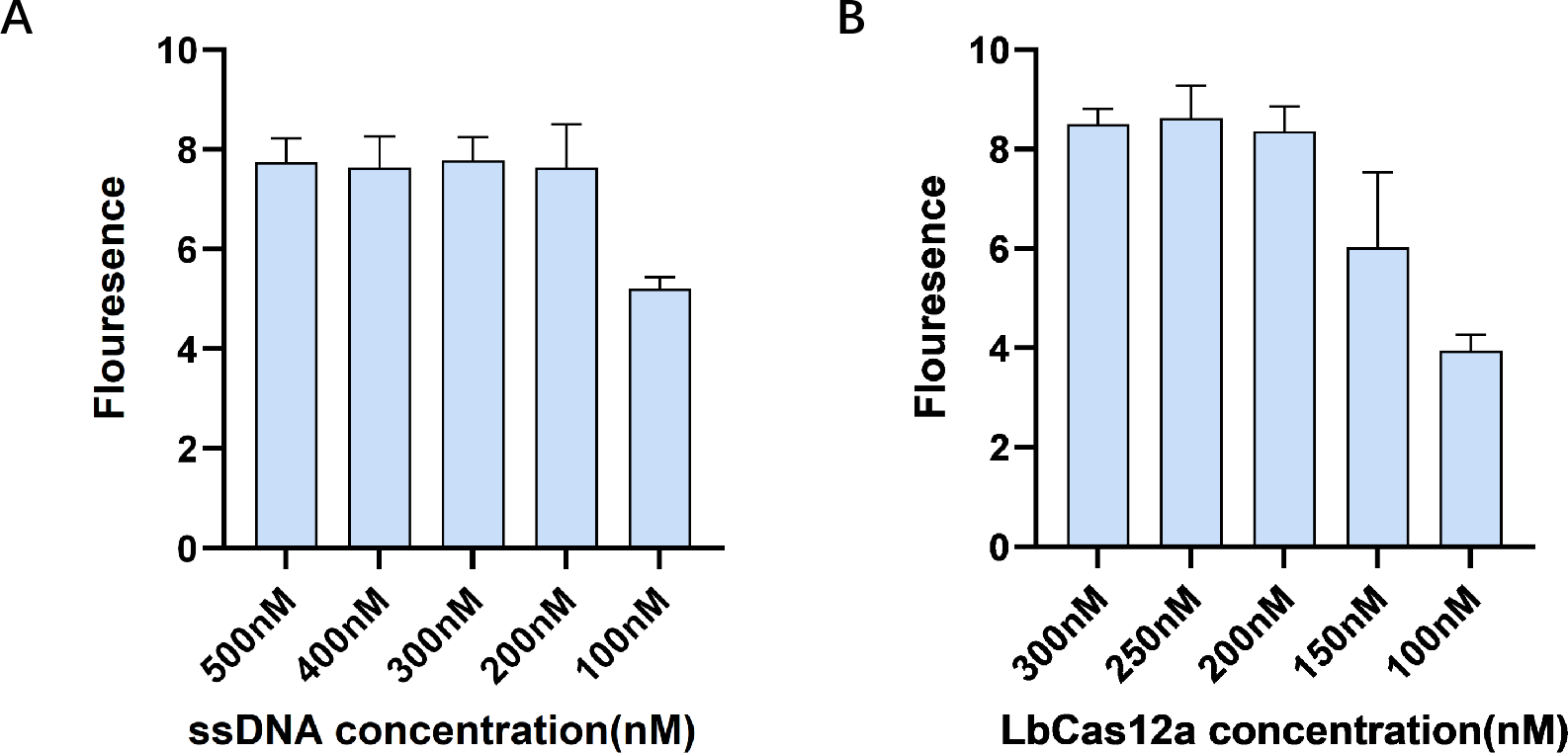
Optimization of the fluorescence detection system based on CRISPR/Cas12a. (**A**) Influence of various ssDNA concentrations on the CRISPR reaction. (**B**) Influence of differing ratios of LbCas12a/crRNA concentrations on the CRISPR reaction. Average results from three biological replicates are displayed.

First, optimization of the ssDNA concentration showed that fluorescence values increased progressively with concentrations rising from 100 nM to 200 nM and saturated at 200 nM. No further increase in fluorescence was observed beyond this concentration, indicating that 200 nM is the optimal concentration for maximum fluorescence intensity (Fig. 3A). Thus, the ssDNA reporter probe concentration was established at 200 nM.

Second, while maintaining the crRNA concentration at 250 nM, the concentration of LbCas12a was varied from 100 nM to 300 nM.

Fluorescence values increased with higher concentrations of LbCas12a and plateaued, identifying 200 nM as the minimal concentration necessary for maximum fluorescence intensity (Fig. 3B). Consequently, the concentration of LbCas12a was set at 200 nM.

### Establishment of a two-step CRISPR/Cas12a method and sensitivity analysis

**Fig 4:**
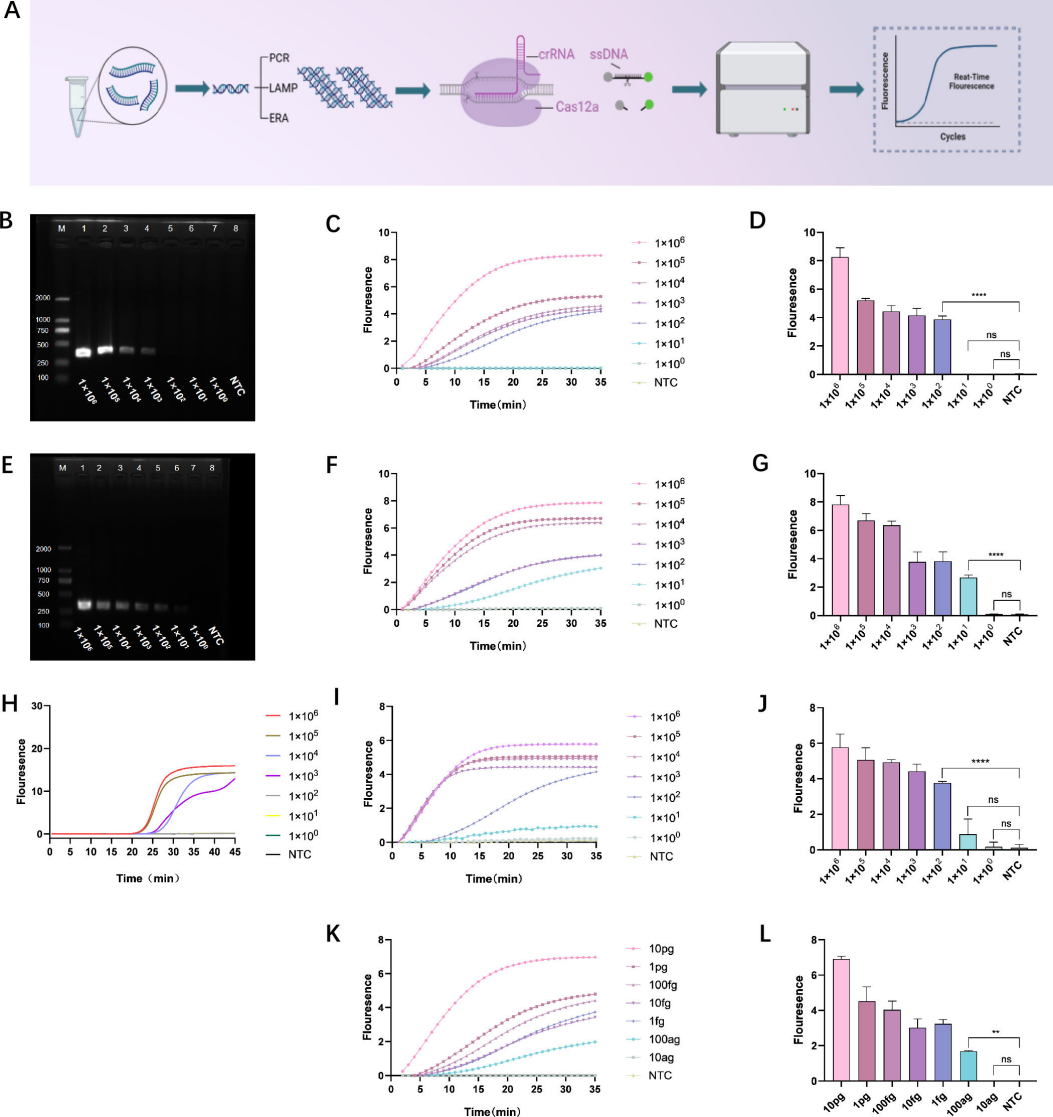
Establishment and sensitivity analysis of the two**-**step CRISPR/Cas12a method. (**A**) Schematic diagram illustrating the workflow for the two**-**step detection of *C. albicans*. (**B**) Analysis of PCR amplification products using agarose gel electrophoresis, utilizing a recombinant plasmid as the template. (**C, D**) Fluorescence curves and fluorescence intensity at 30 minutes for PCR**-**CRISPR/Cas12a, using a recombinant plasmid as the template. (**E**) Analysis of ERA amplification products through agarose gel electrophoresis, utilizing a recombinant plasmid as the template. (**F, G**) Fluorescence curves and fluorescence intensity at 30 minutes for ERA**-**CRISPR/Cas12a, using a recombinant plasmid as the template. (**H**) Monitoring of target LAMP amplification using a dye method, with a recombinant plasmid as the template. (**I, J**) Fluorescence curves and fluorescence intensity at 30 minutes for LAMP**-**CRISPR/Cas12a, using a recombinant plasmid as the template. (**K, L**) Fluorescence curves and fluorescence intensity at 30 minutes for ERA**-**CRISPR/Cas12a, using the genome of pure cultures of *C. albicans* as the template. The results represent the average of three biological replicates, with the negative control (NTC) using nuclease-free water as a substitute (ns: *P* > 0.05; *P < 0.05; **P < 0.01; ***P < 0.001; ****P < 0.0001).

The two**-**step CRISPR/Cas12a detection method for *C. albicans* involves initial amplification of target DNA using three methods—PCR, ERA, and LAMP—followed by integration into the CRISPR/Cas12a system for real-time fluorescence monitoring using a quantitative PCR instrument (Fig. 4A). Sensitivity was first evaluated under single amplification conditions by diluting a recombinant plasmid 10-fold to serve as the template. Agarose gel electrophoresis was performed on products from PCR and ERA to assess sensitivity, while LAMP used a dye method for monitoring nucleic acid amplification.

Subsequently, the combined sensitivity of the amplification and CRISPR fluorescence systems was assessed. The recombinant plasmid was similarly diluted, and following amplification, 2 µL of each product was transferred to the CRISPR system for fluorescence detection. The results demonstrated limits of detection (LOD) of 1 × 10^3^ copies/µL (Fig. 4B and H), and 1 × 10^2^ copies/µL for ERA (Fig. 4E). Based on fluorescence curves and intensity at 30 minutes, the LODs for the two**-**step PCR**-**CRISPR/Cas12a, ERA**-**CRISPR/Cas12a, and LAMP-CRISPR/Cas12a methods were 1 × 10^2^, 1 × 10¹, and 1 × 10^2^ copies/µL, respectively (Fig. 4C and D, F and G, I and J).

The ERA**-**CRISPR/Cas12a method exhibited superior sensitivity. ERA, an isothermal amplification technique, aligns well with the operational temperature of the LbCas12a enzyme, completing within 20 minutes at 37°C–40°C. Consequently, ERA**-**CRISPR/Cas12a was selected for further research in detecting *C. albicans*. This method’s efficacy was validated by testing the genome of pure cultures of *C. albicans*, diluted 10-fold from 10 pg/µL to 10 ag/µL. After ERA amplification, 2 µL of the product was introduced to the CRISPR system for fluorescence assessment. The two**-**step ERA**-**CRISPR/Cas12a method achieved an LOD of 100 ag/µL (Fig. 4K and L), with the entire process**-**amplification plus detection**-**taking about 50 minutes.

### A one-step ERA-CRISPR/Cas12a assay

**Fig 5:**
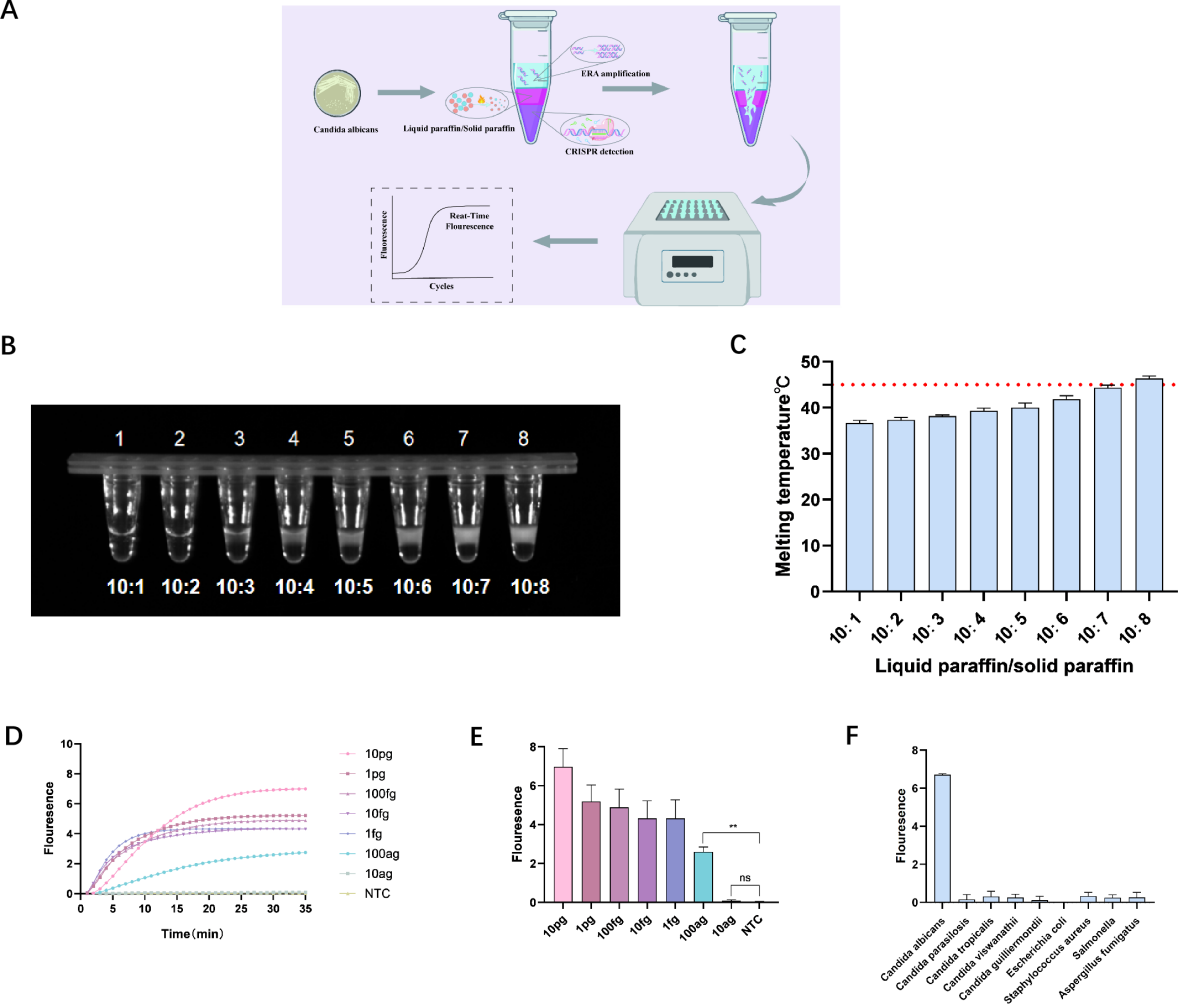
Sensitivity and specificity assessment of the one-step ERA-CRISPR/Cas12a method. (**A**) Schematic diagram of the one-step detection process for *C. albicans*. (**B**) Consistency of various wax mixtures at room temperature. (**C**) Melting temperatures required for different ratios of wax mixtures. (**D, E**) Fluorescence curves and fluorescence intensity at 20 minutes for the one-step ERA**-**CRISPR/Cas12a detection system, using the *C. albicans* genome as a template after initiating the detection reaction. (**F**) Specificity analysis of the one-step ERA-CRISPR/Cas12a method. Average results from three biological replicates are displayed, with negative controls (NTC) using nuclease**-**free water (ns: *P* > 0.05; *P < 0.05; **P < 0.01; ***P < 0.001; ****P < 0.0001).

To optimize experimental procedures and reduce the risk of aerosol contamination from amplicons during lid opening, a one-step ERA**-**CRISPR/Cas12a detection method was developed. This method involves using a wax mixture, composed of liquid and solid paraffin in a specific ratio, to achieve an appropriate melting temperature. This temperature must exceed the amplification temperature but remain within the operational range of LbCas12a. In this setup, ERA amplification reagents and CRISPR/Cas12a detection reagents are separated within the same tube. The process begins by reacting the ERA amplification reagents at 37°C for 10 minutes, followed by an increase to 45°C to melt the wax. Once melted, the amplification reagents mix with the detection reagents and react for 20 minutes, producing corresponding fluorescence signals (Fig. 5A).

Different ratios of liquid to solid paraffin were tested, and their consistency at room temperature was documented (Fig. 5B). The melting temperatures for these mixtures were also recorded using a metal bath (Fig. 5C). It was observed that higher ratios resulted in higher melting temperatures. Based on these results, a ratio of 10:7 of liquid to solid paraffin was selected, setting the melting temperature at 45°C for the experiments. Following the initiation of the detection system, the fluorescence curves and intensity at 20 minutes were analyzed, establishing a one-step ERA**-**CRISPR fluorescence detection limit (LOD) of 100 ag/µL (Fig. 5D and E). The entire process required approximately 40 minutes, demonstrating that the sensitivity of this one-step method was comparable to that of the two-step method while simplifying the workflow and significantly reducing contamination risks. The time from amplification to detection was reduced from 50 to 30 minutes, improving the detection speed.

The specificity of this method was subsequently validated using genomes from various fungi, including *C. albicans* and others such as *C. parapsilosis*, *C. tropicalis*, *C. viswanathii*, *C. guilliermondii*, *E. coli*, *S. aureus*, *Salmonella*, and *Aspergillus fumigatus*. As illustrated in Fig. 5F, only the genome of *C. albicans* produced a strong fluorescence signal, while other genomes showed no significant fluorescence, confirming the absence of cross-reactivity and demonstrating the specificity of the ERA amplification primers.

### Clinical saliva sample detection of one-step ERA CRISPR/Cas12a

**Fig 6:**
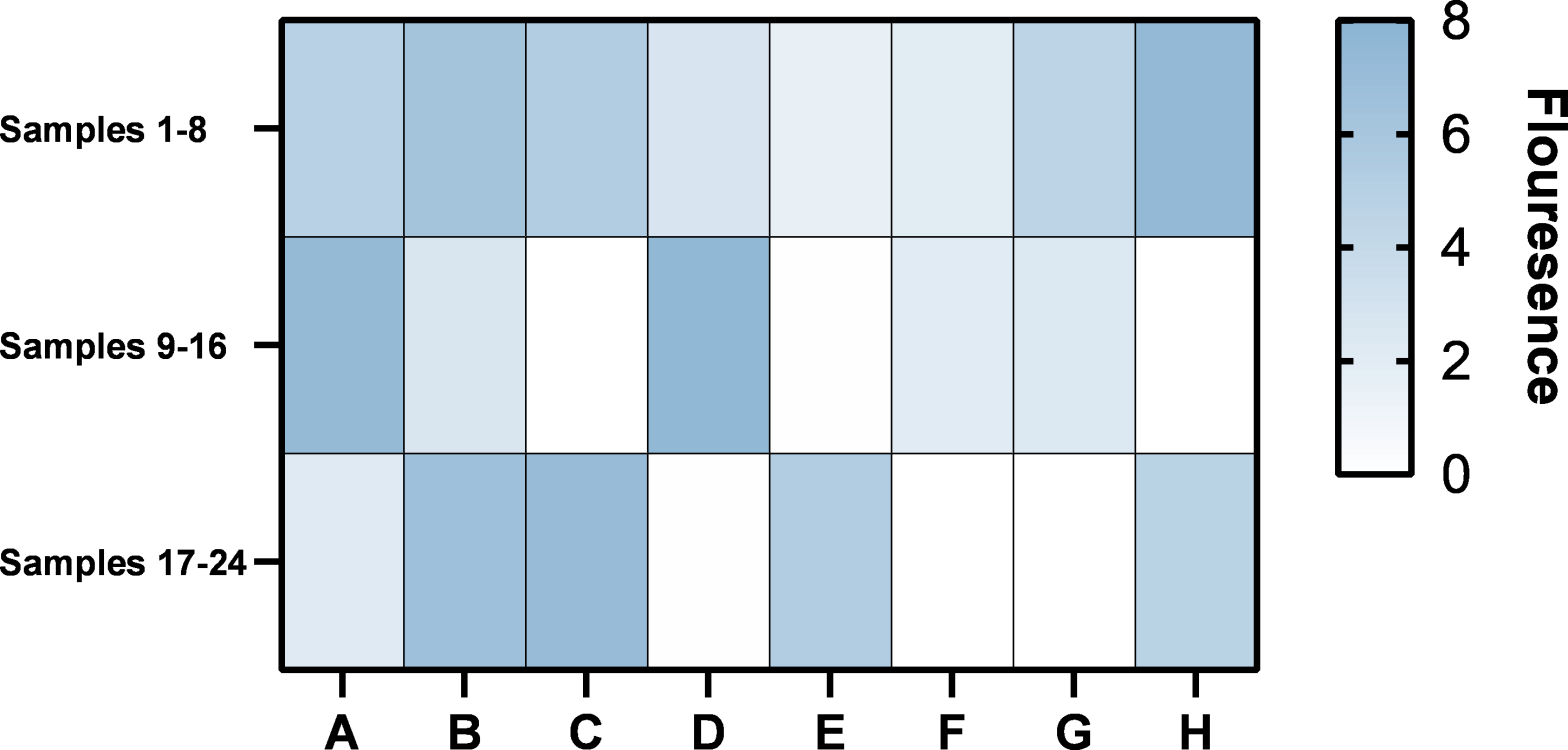
Heatmap of the results obtained from one-step ERA-CRISPR/Cas12a detection of saliva nucleic acid samples.

To validate the clinical applicability of the one-step ERA CRISPR/Cas12a method, the influence of interfering nucleic acids on detection efficacy was assessed. Twenty**-**one negative saliva nucleic acid samples from Huaibei City People’s Hospital were used, with these samples acting as sources of nucleic acid interference. Eighteen were randomly contaminated to create positive samples, with *C. albicans* genome concentrations ranging from 10 pg/µL to 100 ag/µL. The testing involved 18 positive samples, 3 NC, and 3 NTC where nuclease**-**free water substituted for nucleic acids. The concentrations of the 24 samples are listed in Table S2. As depicted in Fig. 6, fluorescence was detected in all eighteen positive samples, but not in the NC and NTC samples. This consistency between the detection results and the contaminated samples demonstrated the method’s high sensitivity, specificity, and accuracy for detecting *C. albicans* in clinical settings, underscoring its significant potential for clinical diagnostics.

### Conclusion

Early identification and treatment are critical for managing candidiasis. A prospective survey conducted in 67 ICU hospitals across China found that the incidence of invasive candidiasis in these settings is 0.32%. Moreover, this disease is an invasive infection in the human body, including bloodstream infection (also known as candidemia) and other deep infections. Among them, bloodstream infection is the most common type, followed by abdominal cavity infection, thoracic cavity infection, etc. Overall, the main pathogen of invasive candidiasis or candidemia is *C. albicans*, accounting for 34.4% to 61.0% (1, 30). Currently, the traditional culture method is still used to identify *C. albicans*, but it has some limitations. The detection cycle is relatively long, and the steps are cumbersome. It requires certain technical skills for the testers, and it is difficult to meet the clinical requirements. Therefore, we are in urgent need of a technology for the rapid diagnosis of *C. albicans*.

In this study, we successfully established a temperature-controlled one-step ERA-CRISPR/Cas12a detection system for *C. albicans* by combining the amplification system and the CRISPR/Cas detection system. This system only needs to be amplified for 10 minutes at 37°C, and the amplification reagent can react with the detection reagent for 20 minutes. The whole process takes 30 minutes. Since the mixed wax is used as the medium in the tube, the fluorescence detection can be carried out in a real-time quantitative fluorescence PCR instrument throughout the amplification and detection process without opening the lid, reducing the aerosol contamination. The detection limit of the one-step *C. albicans* genome LOD can reach 100 ag/µL, which is consistent with the sensitivity of the two-step method. It performs well in detecting its specificity and clinical nucleic acid interference, and there is no cross-reaction phenomenon, showing high sensitivity and specificity.

Therefore, the temperature-controlled one-step ERA-CRISPR/Cas12a system we studied has certain advantages in the detection of *C. albicans* and clinical rapid diagnosis. However, the current detection system still has limitations to be solved. In addition to *C. albicans*, there are four other common Candida species that cause invasive candidiasis: *C. parapsilosis*, *C. glabrata*, *C. tropicalis*, and *C. krusei*. This study only focuses on the detection of *C. albicans*. Relevant improvements will be made in the follow-up, and the application of multiplex nucleic acid detection will be developed to provide assistance for early diagnosis and monitoring.

## SUPPLEMENTAL MATERIAL

Table S1 spectrum.00268-25-s0001.xlsx All the oligonucleotides used in this study.

Table S2 spectrum.00268-25-s0002.docx A list of 24 contaminated nucleic acid samples.

ASM does not own the copyrights to Supplemental Material that may be linked to, or accessed through, an article. The authors have granted ASM a non-exclusive, world-wide license to publish the Supplemental Material files. Please contact the corresponding author directly for reuse.
