# Supplementary material for: Establishment and optimization of a system for the detection of Candida albicans based on enzymatic recombinase amplification and CRISPR/Cas12a system
Source: Microbiol Spectr. 2025 Mar 31;13(5):e00268-25. doi: 10.1128/spectrum.00268-25 (PMC12054178; doi:10.1128/spectrum.00268-25)
Supplement: Table S2 — A list of 24 contaminated nucleic acid samples. [file spectrum.00268-25-s0002.docx]

**Table S2 A List of 24 Contaminated Nucleic Acid Samples**

| 10fg | 10pg | 100fg | 10fg | 100ag | 1fg | 10fg | 1pg |
| --- | --- | --- | --- | --- | --- | --- | --- |
| 10pg | 1fg | NC | 10pg | NTC | 100ag | 1fg | NC |
| 100ag | 1pg | 1pg | NTC | 100fg | NC | NTC | 100fg |
